# The Morphology of the Pituitary Gland: A Meta-Analysis with Implications for Diagnostic Imaging

**DOI:** 10.3390/brainsci13010089

**Published:** 2023-01-02

**Authors:** Michał Bonczar, Grzegorz Wysiadecki, Patryk Ostrowski, Mateusz Michalczak, Dawid Plutecki, Jakub Wilk, Weronika Michalik, Jerzy Walocha, Krzysztof Balawender, Tomasz Iskra, Dariusz Lusina, Mateusz Koziej, Maciej Radek, Andrzej Żytkowski

**Affiliations:** 1 Department of Anatomy, Jagiellonian University Medical College, 33-332 Kraków, Poland; 2 Department of Normal and Clinical Anatomy, Chair of Anatomy and Histology, Medical University of Lodz, 90-752 Łódź, Poland; 3 Collegium Medicum, Jan Kochanowski University, 25-317 Kielce, Poland; 4 Department of Normal and Clinical Anatomy, Institute of Medical Sciences, Medical College of Rzeszow University, 35-315 Rzeszów, Poland; 5 Department of Neurosurgery, Spine and Peripheral Nerves Surgery, Medical University of Lodz, 90-549 Łódź, Poland; 6 Faculty of Philology, Department of Polish Dialectology and Logopedics, University of Lodz, 90-236 Łódź, Poland; 7 Norbert Barlicki Memorial Teaching Hospital No. 1 of the Medical University of Lodz, 90-001 Łódź, Poland

**Keywords:** morphometry, pituitary gland, pituitary fossa, neuroanatomy, neurosurgery

## Abstract

The objective of this meta-analysis was to present transparent data on the morphology of the pituitary gland (PG) using the available data in the literature. The main online medical databases, such as PubMed, Embase, Scopus, and Web of Science, were searched to gather all relevant studies regarding PG morphology. The mean overall volume of the PG was found to be 597.23 mm^3^ (SE = 28.81). The mean overall height of the PG was established to be 5.64 mm (SE = 0.11). The mean overall length of the PG was found to be 9.98 mm (SE = 0.26). In the present study, the PG’s overall morphology and morphometric features were analyzed. Our results showed that, on average, females from Asia have the highest volume of PG (706.69 mm^3^), and males from Europe have the lowest (456.42 mm^3^). These values are crucial to be aware of because they represent the normal average properties of the PG, which may be used as reference points when trying to diagnose potential pathologies of this gland. Furthermore, the present study’s results prove how the PG’s size decreases with age. The results of the present study may be helpful for physicians, especially surgeons, performing procedures on the PG.

## 1. Introduction

The pituitary gland (PG) is a small bean-shaped organ located at the base of the brain. It was termed the “master gland” because of the numerous hormones that emanate from it and help to regulate vital functions such as the growth of various tissues, blood pressure, and reproduction. It is located in the sella turcica, which is the saddle-like bony formation on the upper surface of the body of the sphenoid. The PG consists of the anterior pituitary lobe (or adenohypophysis), the intermediate lobe, and the posterior pituitary lobe (or neurohypophysis). 

During the fourth week of gestation, Rathke’s pouch originates from the roof of the stomodeum. It proliferates to produce the pars distalis (anterior pituitary), the pars intermedia (intermediate lobe), and the pars tuberalis [1,2]. Furthermore, between the fifth and sixth week of gestation, the diencephalon neuroectoderm produces the median eminence, the infundibular stem, and the pars nervosa [3]. 

The “norm” in anatomy is not as precise a concept as one would wish and could be considered an approximation [4]. It is known that pituitary size depends on age, gender, certain diseases, and even race. There is a trend for larger pituitary volumes among females and during puberty, followed by a progressive decrease in size after adolescence [5]. Furthermore, pituitary height was proposed as the best single surrogate parameter of pituitary size and is maximal in young adults and changes with chronic functional alterations [6]. PG volume may also change in patients with various nutritional disorders and neuroendocrine and psychiatric diseases such as schizophrenia, depression, and psychotic disorders [7,8,9,10]. Moreover, morphological changes in the PG due to hormone-related factors, such as the intake of estrogens, pregnancy, and the postpartum phase, have also been a repeated topic of discussion [11,12]. Magnetic resonance imaging (MRI) allows for a detailed examination of PG morphology because of its excellent contrast resolution. Some of the standard methods used in MRI measuring the PG volume are based on stereological techniques using point counting, planimetry, and the elliptic formula [13]. 

Pituitary disorders cause a broad spectrum of hormonal and neurological symptoms due to the gland’s location close to vital neurovascular structures and the essential hormonal control it provides [14]. Therefore, the objective of this meta-analysis was to present transparent data on the morphology of the PG using the available data in the literature. These results could provide physicians with reliable knowledge about the PG’s morphology and morphometry, enabling the recognition of pathological changes in the gland.

## 2. Materials and Methods

### 2.1. Search Strategy

The main online medical databases, such as PubMed, Embase, Scopus, and Web of Science, were searched to gather all relevant studies regarding PG morphology. The following search terms were used: ((pituitary gland) OR ((hypophysis)) AND (anatomy))) OR ((pituitary gland[Title/Abstract]) AND (morphometry[Title/Abstract])). The search terms were adjusted for each database to maximize the number of studies found. The date, language, article type, and text availability conditions were not applied. An additional search was conducted through the references of the identified studies at the end of the search stage to ensure the precision of the process. The preferred reporting items for systematic reviews and meta-analysis (PRISMA) guidelines were followed during the study. The critical appraisal tool for anatomical meta-analysis (CATAM) was also used to provide the highest-quality findings [15]. 

### 2.2. Eligibility Assessment

**Figure 1:**
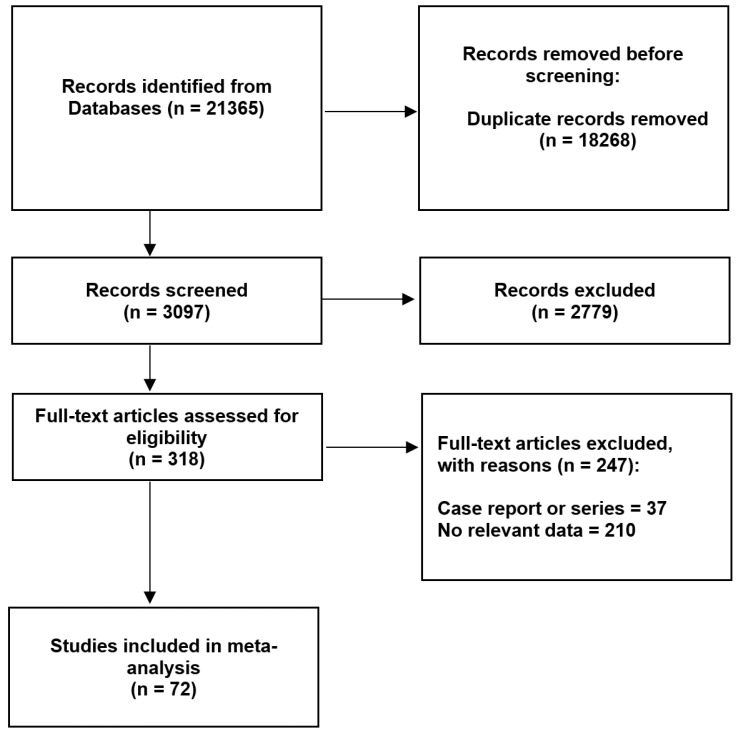
Flow diagram presenting the process of collecting the data included in this meta-analysis.

Initially, after the search of the databases and an additional manual search through the references, 21,365 studies were identified and first reviewed by two independent reviewers. After removing the duplicates and irrelevant records, a total of 318 articles qualified for the full-text evaluation. Papers such as case reports, case series, conference reports, reviews, letters to editors, and studies that provided incomplete or irrelevant data were excluded to minimize potential bias and maintain an accurate statistical methodology. The inclusion criteria involved original studies, both cadaveric and based on radiological imagining, with extractable numerical data on the morphology of the PG. Studies examining the PG in only one dimension were also considered. The results obtained on the cadavers did not differ statistically significantly from those obtained using magnetic resonance imaging (*p* > 0.05); therefore, an overall analysis could be performed. A total of 247 articles were excluded from the study because they were case reports or case series (*n* = 38) or because they did not have relevant or sufficient data regarding the morphometric parameters of the PG (*n* = 210). Finally, a total of 72 studies were included in this meta-analysis. The AQUA Tool, which was specifically designed for anatomical meta-analyses, was used to minimize the potential bias of the included studies [16]. The data collection process is shown in Figure 1. The characteristics of the studies included in this meta-analysis are gathered in Table 1.

### 2.3. Data Extraction

Two independent reviewers performed the extraction. Qualitative data, such as the year of publication, country, and continent, were gathered. Quantitative data, such as the sample size and numerical data regarding the morphological aspects of the PG in specific groups, were gathered. Any discrepancies between the studies identified by the two reviewers were resolved by contacting the authors of the original studies wherever possible or by consensus with a third reviewer.

### 2.4. Statistical Analysis

To perform this meta-analysis, STATISTICA version 13.1 software (StatSoft Inc., Tulsa, OK, USA), MetaXL version 5.3 software (EpiGear International Pty Ltd, Wilston, Queensland, Australia), and Comprehensive Meta-analysis version 3.0 software (Biostat Inc., Englewood, NJ, USA) were applied. A random effects model was used. The Chi-square test and the I-squared statistic were chosen to assess the heterogeneity among the studies [15]. P-values and confidence intervals were used to determine the statistical significance between the studies. A p-value lower than 0.05 was considered statistically significant. In the event of overlapping confidence intervals, the differences were considered statistically insignificant. I-squared statistics were interpreted as follows: values of 0–40% were considered as “might not be important”, values of 30–60% were considered as “might indicate moderate heterogeneity”, values of 50–90% were considered as “may indicate substantial heterogeneity”, and values of 75–100% were considered as “may indicate substantial heterogeneity.” The results obtained on the cadavers did not differ statistically significantly from those obtained using magnetic resonance imaging (*p* > 0.05). Therefore, an overall analysis could be performed.

## 3. Results

### 3.1. Morphometric Parameters of the PG in Adults

The mean overall volume of the PG was 597.23 mm^3^ (SE = 28.81). The mean overall height of the PG was 5.64 mm (SE = 0.11). The mean overall length of the PG was 9.98 mm (SE = 0.26). The mean overall width of the PG was determined at 13.08 mm (SE = 1.76). The mean overall pituitary area was 38.06 mm^3^ (SE = 2.32). The width of the infundibulum was determined at 2.10 mm (SE = 0.07). The most common shape of the PG was convex with a prevalence of 58.38% (CI: 22.21%–90.65%), followed by concave 17.72% (CI: 5.63%–34.00%) and flat 16.67% (CI: 0.00%–47.07%). Partially empty sella turcica occurred with a prevalence of 2.20% (CI: 0.00%–7.16%). The *p*-value in every category equaled < 0.001. All the results mentioned above, and more detailed results, including sexual dimorphisms and intercontinental differences, are gathered in Table 2. 

### 3.2. Morphometric Parameters of the PG in Children (under 18 Years Old)

The mean overall volume of the PG was 511.53 mm^3^ (SE = 112.45). The mean overall height of the PG was 4.81 mm (SE = 0.55). The mean width of the PG was determined at 10.80 mm (SE = 0.07) in Asian children. The mean pituitary area was 29.98 mm^3^ (SE = 1.10) in Asian children. The most frequent shape of the PG was convex, with a prevalence of 54.03% (CI: 22.55%–84.05%), followed by flat 33.10% (CI: 7.87%–64.05%). The prevalence of the concave shape was determined to be 12.16% (CI: 6.92%–18.58%). The *p*-value in every category was < 0.001. All the results mentioned above, and more detailed results, including sexual dimorphisms and intercontinental differences, are gathered in Table 3. 

### 3.3. PG Height concerning the Age Groups of the Patients

**Figure 2:**
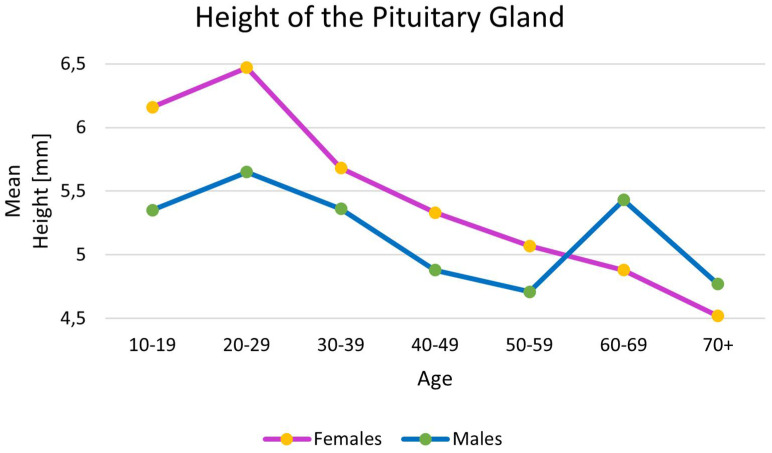
Diagram presenting differences in the heights of the pituitary gland (PG) between male and female patients and different age groups. Statistically significant differences are noted between the females and males in the age group 20-29 (*p* < 0.001).

The results concerning the height of the PG in different age groups were also collected. The detailed statistical data are gathered in Table 4. A graphical representation of the differences in the heights of the PG between male and female patients and between different age groups is displayed in Figure 2.

## 4. Discussion

**Figure 3:**
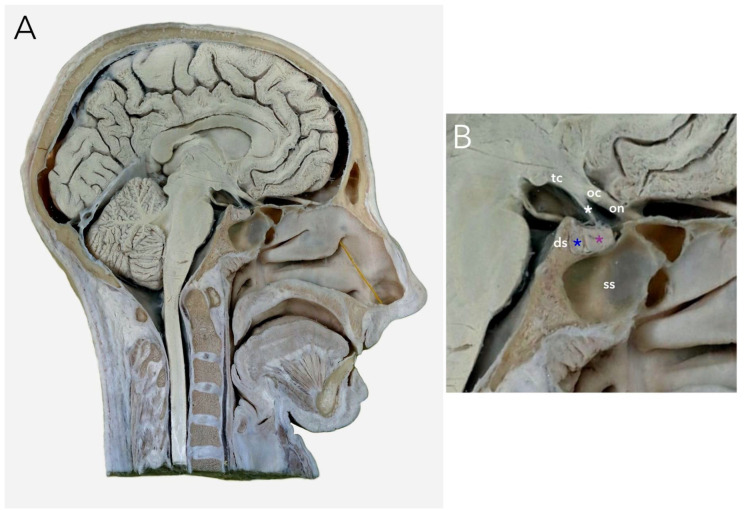
Pituitary gland on the sagittal cross-section of a human cadaver head. (**A**) general view; (**B**) magnification showing the sellar region. ds, dorsum sellae; oc, optic chiasm; on, optic nerve; ss, sphenoid sinus; tc, tuber cinereum; white asterisk, pituitary stalk; violet asterisk, anterior pituitary (front lobe); navy asterisk, posterior pituitary (back lobe).

The PG is a pea-sized gland that sits in the hypophyseal fossa of the sphenoid bone and is surrounded by the sella turcica, covered by the dural fold (diaphragma sellae) (Figure 3). In humans, it comprises two main lobes, anterior and posterior, with the intermediate lobe joining them together. 

During the first two decades of life, the PG grows rapidly and weighs approximately 500 mg by 20 years of age [3,81]. However, with time, significant interstitial fibrosis of the pituitary tissue develops, leading to decreased proportions of the gland [81]. Furthermore, the PG goes through considerable changes in size during pregnancy. It was proven that during pregnancy, the PG becomes somewhat hyperintense on T1-weighted images (one of the basic pulse sequences in MRI that demonstrates the differences in the T1 relaxation times of tissues), similar to that described for neonates [81,82]. 

The morphological properties of the PG vary considerably in the general population. Singh et al. conducted a study analyzing the morphometry of the PG using MRI [24]. In the study, the authors stated that the differences in the mean pituitary height and volume between females and males were statistically significant. The mean PG height was reported to be 5.80 ± 1.32 mm and 5.37 ± 1.25 mm for females and males, respectively. Another study by Suzuki et al. presented lower results concerning the height of the PG (5.0 ± 1.7 mm in women and 4.7 ± 1.4 mm in men) [74]. Denk et al. [83], in turn, reported higher values than those in the studies mentioned above (6.1 ± 0.1 mm in women and 5.6 ± 0.2 mm in men). Although the PG height varies between the studies, on average, it is greater in females than in males. Our meta-analysis provides similar results, where females had higher PGs than males (5.40 ± 0.14 mm in women and 4.96 ± 0.12 mm in males). 

In the present study, we highlight how the height of the PG changes throughout life. On average, women had taller PGs. However, in females and males, the height peaked in the age group of 20 to 29. With time, the height decreased substantially in females and males, proving that the PG decreases in size throughout life. However, some studies have reported an increase in pituitary height again during the fifth decade of life, especially in females [24,61,67]. The increased activity of gonadotrophs explained this phenomenon in older women due to the loss of negative feedback by gonadal steroids [24]. However, Singh et al. presented an increase in the height of the PG in males [24]. Unfortunately, the mechanism behind this phenomenon is still unknown. However, it may be similar to the one in females, though the phenomenon of andropause in males is not as well understood as menopause in females. Interestingly, our meta-analysis showed that the increase in the height of the PG after the fifth decade of life happened in males only. Furthermore, the average volume of the PG was also analyzed in different populations. Interestingly, the largest PG volume was found to be present in females from Asia (706.69 mm^3^), and the lowest was found in males from Europe (456.42 mm^3^). These values are crucial to be aware of because they represent the typical average properties of the PG, which may be used as reference points when trying to diagnose potential pathologies of this gland. 

Knowledge of the anatomy of the PG and its morphometric properties is of immense importance when performing surgical procedures on it or in the area of the sella turcica. Pituitary adenomas account for approximately 10–15% of surgically treated central nervous system tumors [84]. The standard approach to the pituitary area is the transsphenoidal midline route, which avoids brain retraction and is known to be less traumatic. In this approach, endoscopes are commonly used as the sole visualizing tool. However, if the pituitary tumor has considerable suprasellar and parasellar extension, the transcranial approach might be more suitable [85]. 

The PG may be the subject of numerous incidental and benign conditions. These include cysts, which are incidentally found in approximately 10% to 20% of the population [86], rounded calcifications in the gland, or pituitary stones, that do not present as clinically significant findings [3]. A somewhat controversial pathology of the PG is the empty sella. This phenomenon was first described in the 1950s [87]. It is believed to be caused by a weakening of the diaphragma sella. This, in turn, enables the transmission of the cerebrospinal fluid pulsations in the suprasellar cistern to the intrasellar area [3]. This ultimately leads to an incomplete or complete flatting of the PG and, with that, a loss of physiological functioning of the gland. 

The relation between the morphology of the PG and various degenerative pathologies, such as Alzheimer’s disease (AD), has been a topic of interest in the literature. One protein that is said to play a role in the pathogenesis of AD and Creutzfeldt-Jakob disease is clusterin [88]. Clusterin, or apolipoprotein J, is a circulating glycoprotein with numerous functions, such as lipid transport and immune modulation [89]. An age-dependent increase of clusterin in the PG was studied by Ishikawa et al. [90] in an immunohistochemical study. They found a positive correlation between the age of the subjects in years and the percentage of clusterin-positive cell area in the anterior lobe of the PG. However, there was no statistical relationship between the clusterin levels and gender. These results suggest that PG clusterin is produced following degenerative changes.

However, the mechanism of this phenomenon is still unclear. The role of the hypothalamic-pituitary axis in Alzheimer’s disease was also discussed previously. Interestingly, there is a gender-biased predisposition towards females specific to AD [90]; therefore, the role of sex steroids regulated by the hypothalamus-pituitary-gonadal axis was investigated. Evidence has suggested that estrogen deficiency, which follows menopause, may contribute to the pathogenesis of AD [91]. Estrogen synthesis and secretion from the theca interna cells in the ovary are mainly stimulated by the luteinizing hormone produced in the anterior PG. Therefore, potential pathologies of the PG, such as hypopituitarism, may cause a decrease in the synthesis and secretion of luteinizing hormone and, subsequently, cause decreased estrogen levels. This mechanism may potentially lead to an increased risk of developing AD. Hypopituitarism is most commonly caused by pituitary adenomas, which may be diagnosed with imaging studies. Our study may be helpful for physicians diagnosing the previously mentioned neoplasms because we provide standard morphometric data of the PG, which may be used as the reference point when comparing measured values of a PG with an adenoma. 

Thyroid dysfunction could be a potential risk factor for dementia development; numerous epidemiological data implicates both hyperthyroidism and hypothyroidism [92,93]. Yong-Hong et al. [93] conducted a study where the hypothalamic-pituitary-thyroid axis was assessed. The study showed that the patients with AD had significantly lower thyroid-releasing (TRH) and thyroid-stimulating (TSH) hormones, total and free triiodothyronine, and total and free tetraiodothyronine when compared to the control group. These results present how AD may be associated with the lowered function of the hypothalamic-pituitary-thyroid axis.

The present study is not without limitations. It may be burdened with potential bias, as the accuracy of the data taken from various publications limits the results of this meta-analysis. Additionally, most of the evaluated studies come from Asia. The total number of PG’s analyzed from Asia equaled 1203, while from Europe, there were 1186, and from North America, there were 1093. Therefore, the overall results of this study may be burdened, as they may reflect the anatomical features of Asian people rather than the global population. Variations of organs’ shape and dimensions are frequently observed by medical professionals and could influence daily clinical practice, including imaging diagnostics, therapeutic decisions, and surgical procedures [4]. Although not without limitations, our meta-analysis attempts to estimate pituitary morphology based on the data from the literature that meet the requirements of evidence-based anatomy.

## 5. Conclusions

The present study analyzed the morphology and morphometric features of the PG. Our results show that, on average, females from Asia have the highest volume of PG (706.69 mm^3^), and males from Europe have the lowest (456.42 mm^3^). These values are crucial to be aware of because they represent the normal average properties of the PG, which may be used as reference points when trying to diagnose potential pathologies of this gland. Furthermore, the present study’s results prove how the PG’s size decreases with age. The results of the present study may be helpful for physicians, especially surgeons, performing procedures on the PG.

## Figures and Tables

**Table 1:** Characteristics of the studies included in this meta-analysis.

First Author	Year	Continent	Country	Method	Patients
**Gurok et al.** [17]	2019	Asia	Turkey	MRI	18
**Polat et al.** [18]	2020	Asia	Turkey	MRI	292
**Whittle et al.** [19]	2020	Australia	Australia	MRI	409
**Atmaca et al.** [20]	2018	Asia	Turkey	MRI	12
**Dumrongpisutikul et al.** [21]	2018	Asia	Thailand	MRI	70
**Bozkurt Koseoglu and Dinc Elibol** [22]	2018	Asia	Turkey	MRI	42
**Premkumar et al.** [23]	2018	Europe	UK	MRI	30
**Singh et al.** [24]	2018	Asia	India	MRI	482
**Pecina et al.** [11]	2017	Europe	Croatia	MRI	199
**Muneuchi et al.** [25]	2018	Asia	Japan	MRI	40
**Atmaca et al.** [26]	2016	Asia	Turkey	MRI	20
**Unlu et al.** [27]	2015	Asia	Turkey	MRI	31
**Clark et al.** [28]	2014	Europe	England	MRI	74
**Pieper et al.** [29]	2013	Europe	Germany	MRI	16
**Takahashi et al.** [30]	2013	Asia	Japan	MRI	86
**van der Plas E. et al.** [31]	2012	North America	USA	MRI	279
**Gruner et al.** [7]	2012	North America	USA	MRI	59
**Yildirim et al.** [32]	2012	Asia	Turkey	MRI	18
**Kartalci et al.** [33]	2011	Asia	Turkey	MRI	27
**Klomp et al.** [8]	2012	Europe	Netherlands	MRI	156
**Takahashi et al.** [34]	2011	Asia	Japan	MRI	40
**Atmaca et al.** [35]	2010	Asia	Turkey	MRI	20
**Büschlen et al.** [36]	2011	Europe	Switzerland	MRI	20
**Ertekin et al.** [13]	2011	Asia	Turkey	MRI	28
**Grams et al.** [37]	2010	Europe	Germany	MRI	94
**Nicolo et al.** [10]	2010	Australia	Australia	MRI	48
**Takahashi et al.** [38]	2010	Europe	UK	MRI	52
**Atmaca et al.** [39]	2009	Asia	Turkey	MRI	23
**Garner et al.** [40]	2009	Oceania	Australia	MRI	42
**Jung et al.** [41]	2009	Asia	South Korea	MRI	62
**Lorenzetti et al.** [42]	2009	Oceania	Australia	MRI	33
**Takahashi et al.** [43]	2009	Australia	Australia	MRI	122
**Eker et al.** [44]	2008	Asia	Turkey	MRI	39
**Jovev et al.** [45]	2008	Oceania	Australia	MRI	20
**Miranda-Scippa et al.** [46]	2008	South America	Brazil	MRI	24
**Unlu et al.** [47]	2008	Asia	Turkey	Cadavers	5
**Yilmazlar et al.** [48]	2008	Asia	Turkey	Cadavers	49
**Garner et al.** [49]	2007	Oceania	Australia	MRI	20
**Gong et al.** [50]	2007	North America	Canada	Cadavers	42
**Upadhyaya et al.** [51]	2007	North America	USA	MRI	55
**Garner et al.** [52]	2005	Oceania	Australia	MRI	49
**Miki et al.** [12]	2005	Asia	Japan	MRI	13
**Argyropoulou et al.** [53]	2004	Europe	Greece	MRI	70
**Bolu et al.** [54]	2004	Asia	Turkey	MRI	49
**Chen et al.** [5]	2004	North America	USA	MRI	21
**Macmaster et al.** [9]	2004	North America	Canada	MRI	17
**Kornreich et al.** [55]	2003	Asia	Isreal	MRI	9
**Sassi et al.** [56]	2001	North America	USA	MRI	34
**Takano et al.** [57]	1999	Asia	Japan	MRI	199
**Dinç et al.** [58]	1998	Europe	Denmark	MRI	18
**Sato et al.** [59]	1997	North America	USA	MRI	12
**Schwartz et al.** [60]	1997	North America	USA	MRI	19
**Tsunoda et al.** [61]	1997	Asia	Japan	MRI	1020
**Desai et al.** [62]	1996	Asia	India	MRI	10
**Chong et al.** [63]	1994	North America	Canada	MRI	52
**Teoh et al.** [64]	1993	North America	USA	MRI	8
**Axelson et al.** [65]	1992	Europe	Ireland	MRI	24
**Cox** [66]	1991	North America	USA	MRI	48
**Doraiswamy et al.** [67]	1992	North America	USA	MRI	85
**Elster et al.** [68]	1991	North America	USA	MRI	30
**Krishnan et al.** [69]	1991	North America	USA	MRI	19
**Konishi et al.** [70]	1990	Asia	-	MRI	101
**Argyropoulou et al.** [71]	1991	Europe	France	MRI	60
**Lurie et al.** [72]	1990	North America	USA	MRI	25
**Murali et al.** [73]	1990	North America	USA	MRI	13
**Suzuki et al.** [74]	1990	Asia	Japan	MRI	213
**Gonzalez et al.** [75]	1988	North America	Mexico	MRI	20
**Lim et al.** [76]	1986	Asia	South Korea	CT	11
**Wiener et al.** [77]	1985	North America	USA	MRI	42
**Mark et al.** [78]	1984	North America	USA	MRI	38
**Peyster et al.** [79]	1986	North America	USA	CT	27
**Muhr et al.** [80]	1981	Europe	Sweden	MRI	205

**Table 2:** Results of this meta-analysis regarding the morphometric parameters of the pituitary gland (PG) in adults. LCI, lower confidence interval; HCI, higher confidence interval; Q, Cochran’s Q.

Category	*N*	Mean	Standard Error	Variance	Lower Limit	Upper Limit	Z-Value
**Volume of the PG (mm^3^)**							
Overall	1349	597.23	28.81	830.09	540.76	653.70	20.73
Females	508	587.36	41.76	1744.19	505.50	669.21	14.06
Males	494	508.87	27.95	781.08	454.09	563.64	18.21
Asia	430	669.60	71.89	5168.50	528.69	810.50	9.31
Asian Females	84	706.69	110.26	12,157.12	490.59	922.80	6.41
Asian Males	123	642.04	53.26	2836.19	537.66	746.42	12.06
Australia	81	634.58	74.63	5570.01	488.30	780.86	8.50
Australian Females	38	591.62	43.76	1914.86	505.85	677.38	13.52
Australian Males	43	531.56	34.46	1187.23	464.03	599.09	15.43
Europe	515	562.51	33.39	1115.23	497.06	627.96	16.84
European Females	319	620.09	87.98	7740.12	447.66	792.53	7.05
European Males	196	456.42	23.49	551.59	410.39	502.46	19.43
North America	279	516.59	45.89	2106.05	426.64	606.54	11.26
North American Females	44	584.53	108.61	11,795.77	371.66	797.40	5.38
North American Males	46	535.09	93.79	8797.21	351.26	718.92	5.70
South America	24	626.91	28.10	789.86	571.82	681.99	22.31
**Height of the PG (mm)**							
Overall (MRI + cadavers)	787	5.64	0.11	0.01	5.41	5.86	49.62
MRI	712	5.35	0.14	0.02	5.07	5.63	37.92
Cadavers	75	5.74	0.20	0.04	5.35	6.13	28.60
Females	342	5.40	0.14	0.02	5.13	5.68	38.31
Males	171	4.96	0.12	0.01	4.73	5.18	43.05
Asia	120	6.03	0.47	0.22	5.11	6.96	12.82
Asian Females	38	4.92	0.08	0.01	4.76	5.08	61.94
Asian Males	82	4.70	0.12	0.01	4.47	4.92	40.55
Europe	317	5.75	0.21	0.05	5.33	6.17	26.98
North America	379	5.55	0.16	0.03	5.24	5.86	34.73
North American Females	62	5.59	0.35	0.12	4.90	6.28	15.87
North American Males	51	4.91	0.51	0.26	3.91	5.91	9.62
**Length of the PG (mm)**							
Overall	363	9.98	0.26	0.07	9.46	10.49	37.89
Females	120	10.03	0.34	0.11	9.37	10.69	29.83
Males	133	9.83	0.46	0.21	8.92	10.73	21.36
Asia	77	9.38	0.91	0.83	7.60	11.16	10.31
Europe	112	10.04	0.34	0.11	9.38	10.70	29.81
North America	174	10.13	0.34	0.11	9.47	10.79	29.99
**Width of the PG (mm)**							
Overall (MRI + cadavers)	377	13.08	1.76	3.10	9.63	16.53	7.43
MRI	302	13.18	0.72	0.52	11.76	14.59	18.24
Cadavers	75	15.26	1.37	1.87	12.57	17.94	11.14
Females	294	13.14	0.27	0.07	12.60	13.67	48.46
Males	83	12.91	0.26	0.07	12.39	13.43	48.77
Asia	82	17.53	1.47	2.17	14.64	20.41	11.90
Europe	112	13.92	0.37	0.13	13.20	14.63	37.96
North America	183	12.71	0.59	0.35	11.55	13.86	21.56
**Pituitary Area (mm^2^)**							
Overall	154	38.06	2.32	5.40	33.51	42.62	16.38
Females	32	41.59	6.57	43.23	28.70	54.48	6.33
Males	16	36.00	1.75	3.06	32.57	39.43	20.57
**Infundibulum (mm)**							
Width	18	2.10	0.07	0.01	1.96	2.24	29.70
Category	N	Pooled Prevalence		LCI	HCI	Q	I^2^
**Shape of the PG**							
Convex	309	58.38%		22.21%	90.65%	171.49	95.33
Concave	309	17.72%		5.63%	34.00%	43.95	81.80
Flat	309	16.67%		0.00%	47.07%	135.91	94.11
Partially empty sella	309	2.20%		0.00%	7.16%	16.32	50.97

**Table 3:** Results of this meta-analysis regarding the morphometric parameters of the pituitary gland (PG) in children (under 18 years old). LCI, lower confidence interval; HCI, higher confidence interval; Q, Cochran’s Q.

Category	*N*	Mean	Standard Error	Variance	Lower Limit	Upper Limit	Z-Value
**Volume of the PG (mm^3^)**							
Overall	212	511.53	112.45	12,644.01	291.14	731.92	4.55
Females	101	450.88	48.90	2391.31	355.03	546.72	9.22
Australian Females	32	349.18	7.68	58.93	334.14	364.23	45.49
Males	111	453.50	46.52	2164.44	362.32	544.69	9.75
Australian Males	46	365.04	30.37	922.64	305.50	424.57	12.02
North America	78	513.86	155.43	24,158.38	209.23	818.50	3.31
**Height of the PG (mm)**							
Overall	341	4.81	0.55	0.30	3.73	5.89	8.72
Females	16	6.60	0.50	0.25	5.62	7.58	13.20
Males	16	5.40	0.53	0.28	4.37	6.43	10.29
Asia	211	5.37	0.24	0.06	4.90	5.84	22.37
Europe	130	3.94	0.67	0.45	2.62	5.25	5.85
**Width of the PG (mm)**							
Asia	40	10.80	0.07	0.01	10.66	10.94	151.79
**Pituitary Area (mm^2^)**							
Asia	101	29.98	1.10	1.20	27.83	32.13	27.32
**Category**	**N**	**Pooled Prevalence**		**LCI**	**HCI**	**Q**	**I^2^**
**Shape of the PG**							
Convex	154	54.03%		22.55%	84.05%	38.15	92.14
Concave	154	12.16%		6.92%	18.58%	3.53	14.97
Flat	154	33.10%		7.87%	64.05%	35.25	91.49

**Table 4:** Results of this meta-analysis regarding the height of the pituitary gland (PG) with respect to the age groups of the patients.

Category	Mean	Standard Error	Variance	Lower Limit	Upper Limit	Z-Value	*p*-Value
Height of the PG (mm)							
Females 10–19 years old	6.16	0.23	0.05	5.72	6.61	27.09	<0.001
Males 10–19 years old	5.35	0.30	0.09	4.77	5.93	18.10	<0.001
Females 20–29 years old	6.47	0.11	0.01	6.25	6.68	58.47	<0.001
Males 20–29 years old	5.65	0.13	0.02	5.39	5.90	43.31	<0.001
Females 30–39 years old	5.68	0.17	0.03	5.36	6.01	33.92	<0.001
Males 30–39 years old	5.36	0.14	0.02	5.08	5.65	37.43	<0.001
Females 40–49 years old	5.33	0.23	0.05	4.88	5.77	23.61	<0.001
Males 40–49 years old	4.88	0.10	0.01	4.68	5.08	48.40	<0.001
Females 50–59 years old	5.07	0.42	0.17	4.26	5.89	12.17	<0.001
Males 50–59 years old	4.71	0.17	0.03	4.39	5.04	28.41	<0.001
Females 60–69 years old	4.88	0.09	0.01	4.71	5.06	54.94	<0.001
Males 60–69 years old	4.53	0.29	0.08	3.97	5.10	15.81	< 0.001
Females 70+ years old	4.52	0.47	0.22	3.61	5.43	9.69	<0.001
Males 70+ years old	4.77	0.10	0.01	4.59	4.96	49.83	<0.001

## Data Availability

The data presented in this study are available upon request from the corresponding author.
